# A bibliometric analysis of the application of imaging in sleep in neurodegenerative disease

**DOI:** 10.3389/fnagi.2023.1078807

**Published:** 2023-02-02

**Authors:** Mengfei Li, Zhenzhen Jiang, Ru Wen, Chen Liu, Jian Wang

**Affiliations:** ^1^Department of Radiology, Southwest Hospital, Army Medical University (Third Military Medical University), Chongqing, China; ^2^Department of Medical Information Engineering, College of Medicine, Guizhou University School, Guiyang, Guizhou Province, China

**Keywords:** sleep, imaging, bibliometrics, CiteSpace, degenerative disease

## Abstract

**Objective:**

The purpose of this study was to examine the current state of the application of imaging in sleep research in degenerative disease, as well as hotspots and trends.

**Materials and methods:**

A search was conducted on the Web of Science Core Collection (WoSCC) between 1 September 2012, and 31 August 2022 for literature related to sleep imaging. This study analyzed 7,679 articles published in this field over the past 10 years, using CiteSpace to analyze tendencies, countries, institutions, authors, and hotspots.

**Results:**

There were 7,679 articles on the application of imaging to sleep research published by 566 institutions located in 135 countries in 1,428 journals; the number of articles was increasing on a yearly basis. According to keyword analysis, the research direction of the application of imaging in sleep research focused on the effects of degenerative diseases on sleep, such as Parkinson’s disease, Alzheimer’s disease, and small vessel disease. A literature evaluation found that Parkinson’s disease, insomnia, sleep quality, and rapid eye movement sleep behavior disorder were the top research trends in this field.

**Conclusion:**

A growing body of research has focused on sleep disorders caused by degenerative diseases. In the application of imaging to sleep research, magnetic resonance functional brain imaging represents a reliable research method. In the future, more aging-related diseases may be the subject of sleep-related research, and imaging could provide convenient and reliable evidence in this respect.

## 1. Introduction

Humans sleep most of their lives, but the functional role of sleep remains mysterious. The importance of sleep for the maintenance of many physiological functions, including cognitive function, has become increasingly apparent over the last few decades (Malkani and Zee, 2022). Current studies illustrate that sleep disorders constitute a health burden for all societies (Spaggiari et al., 2022; Xu et al., 2022); many diseases are associated with sleep disorders, including high blood pressure, mental illness, neurodegenerative diseases, and cardiovascular disease, and sleep disorders affect 40–50% of the world’s population (Bin Heyat et al., 2021). Therefore, it is essential to study sleep. Sleep cannot be attributed to any one specific organ, unlike other physiological functions; thus, the study of the functional role of sleep is a systematic and complex task requiring different techniques to reveal its underlying mechanisms. With the development of the disciplines of physiology and psychology, as well as increasing understanding of the structure and function of the nervous system, sleep research has become increasingly objective. Electroencephalography (EEG) became the central monitoring tool in sleep research in the 1930s, to observe the sleeping brain with high temporal resolution (Schulz, 2022). With the rapid development of brain imaging technology, functional imaging has made it possible to image brain activity during sleep at a high spatial resolution (Ferini-Strambi et al., 2019). Compared with functional magnetic resonance imaging, magnetoencephalography is not affected by age-related changes in vascular factors and allows simpler and more powerful methods to correct head motion artifacts (Tibon and Tsvetanov, 2021). Polysomnography is a procedure for evaluating the root causes of sleep disorders using electroencephalogram, electro-oculogram, electrocardiogram, pulse oximeter, airflow, and breathing efforts (Rundo and Downey 3rd, 2019). It is an authoritative and objective tool for studying sleep. Currently, neuroimaging methods play a crucial role in sleep research, both in basic research and clinical fields (Bourgouin et al., 2019).

With the aggravation of the aging population, the problem of sleep to human beings is becoming more and more serious. Sleep imaging research develops over time, and it is vital to explore the hotspots and trends of future applications. Summarizing the experience of prior researchers is helpful to promote more in-depth and valuable research in the future. Bibliometrics is a visual analysis tool that permits quantitative analysis of the literature using mathematical and statistical methods (Glanzel, 2015). It identifies influential and valid areas of scientific research, knowledge bases, and emerging topics (Hou et al., 2018).

This study used CiteSpace (Hou et al., 2018; Chen and Song, 2019) to analyze the literature related to sleep. Cataloged in the Web of Science Core Collection (WoSCC) database, to explore the research status, hotspots, and trends in the application of imaging to sleep in degenerative disease and to provide new perspectives and paths for the study of the application of imaging in sleep research in degenerative disease using imaging.

## 2. Materials and methods

### 2.1. Data retrieval strategy

**Figure 1:**
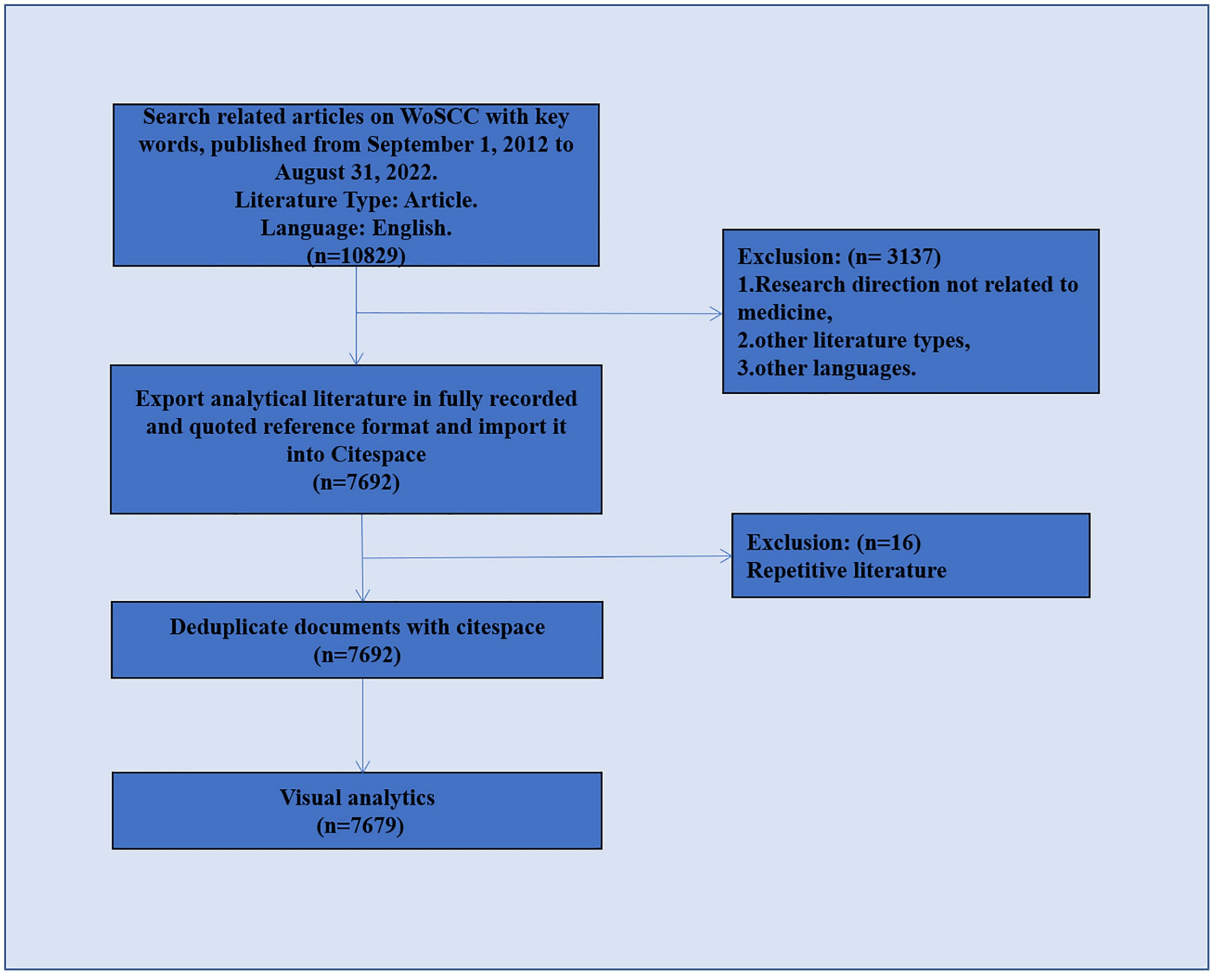
Flow chart of data preparation.

This was a retrospective cross-sectional study regarding imaging of sleep disorders in degenerative disease. Data retrieval was based on the Web of Science Core Collection (WoSCC) database. The WOSCC is a standardized online database that is considered to be the most suitable for bibliometric analysis (Schulz, 2022). To ensure the accuracy of the data, the literature search was completed in 1 day (22 September 2022), with 1 September 2012, to 31 August 2022, selected as the time frame for this study. Since the set time span did not include the literature for the first 8 months of 2012 and the last 4 months of 2022, the data for 2012 and 2022 are incomplete; the analysis does not represent the whole of these 2 years. The primary type of literature selected in this study was articles, and English was the primary language. To identify any possible selection differences, two researchers independently searched the raw data. Figure 1 shows the detailed filtering process.

The search terms were as follows: (TS) = (imaging OR “magnetic resonance imaging” OR MRI OR “functional magnetic resonance imaging” OR fMRI OR “diffusion tensor imaging” OR DTI OR “single-photon emission computed tomography” OR SPECT OR “positron emission tomography” OR PET OR “structural neuroimaging” OR neuroimaging) AND TS = (“insomnia disorder” OR insomnia OR “REM behavior sleep disorder” OR “excessive daytime sleepiness” OR sleep).

### 2.2. Data export and extraction

CiteSpace^1^ was used to analyze collaborative networks (country/region, institution, author, and journal), co-citations (author, journal, and reference), and co-occurrence bursts of keywords. CiteSpace (Chen and Song, 2019) 6.1.R2 parameters were set as follows: time slice, August 2012 to September 2022, 1 year per slice; text processing, title, abstract, author, keywords; node type, from country/region, institution, author, keyword, co-cited journal, co-cited author, and co-cited literature; link strength, cosine; link range, within slices; selection criteria, *g*-index, *k* = 25; pruning, pathfinding network method and pruning slices, integrated network; using the pathfinding network algorithm. Statistics were imported into Microsoft Office Excel 2019^2^ for graphing.

In CiteSpace, the “*g*-index” is used as a selection criterion. This index is defined as the largest number whose sum of citations is at least *g*-squared. If papers are listed in descending order of their citations, highly cited articles are more accurately reflected by this index than by the H-index (Abbas, 2012). In CiteSpace, *k* is a scale factor that is added to the *g*-index calculation and adjusted to include or exclude more nodes (Abbas, 2012). “Burst detection” and “betweenness centrality” are features of the software that allow researchers to identify new trends, research frontiers, and abrupt changes in a research area. Betweenness centrality is a measure of the importance of nodes in a network and is guided by tree hole theory (Assenov et al., 2008; Chen et al., 2014). Additionally, literature co-citation refers to the relationship that exists between two (or more) papers that are cited simultaneously by one or more subsequent papers (Ding et al., 2021). In this study, the algorithm was used to extract noun phrases (Chen and Song, 2019). During the analysis, we assessed network structure and network homogeneity based on modules (Q-value) and profiles (S-value). Q-values greater than 0.3 indicate significant clustering structure, while S-values greater than 0.7 indicate high clustering confidence (Sabe et al., 2022). By examining these parameters, we determined the state of the research and trends in the field.

## 3. Results

### 3.1. Publication outputs and trends

**Figure 2:**
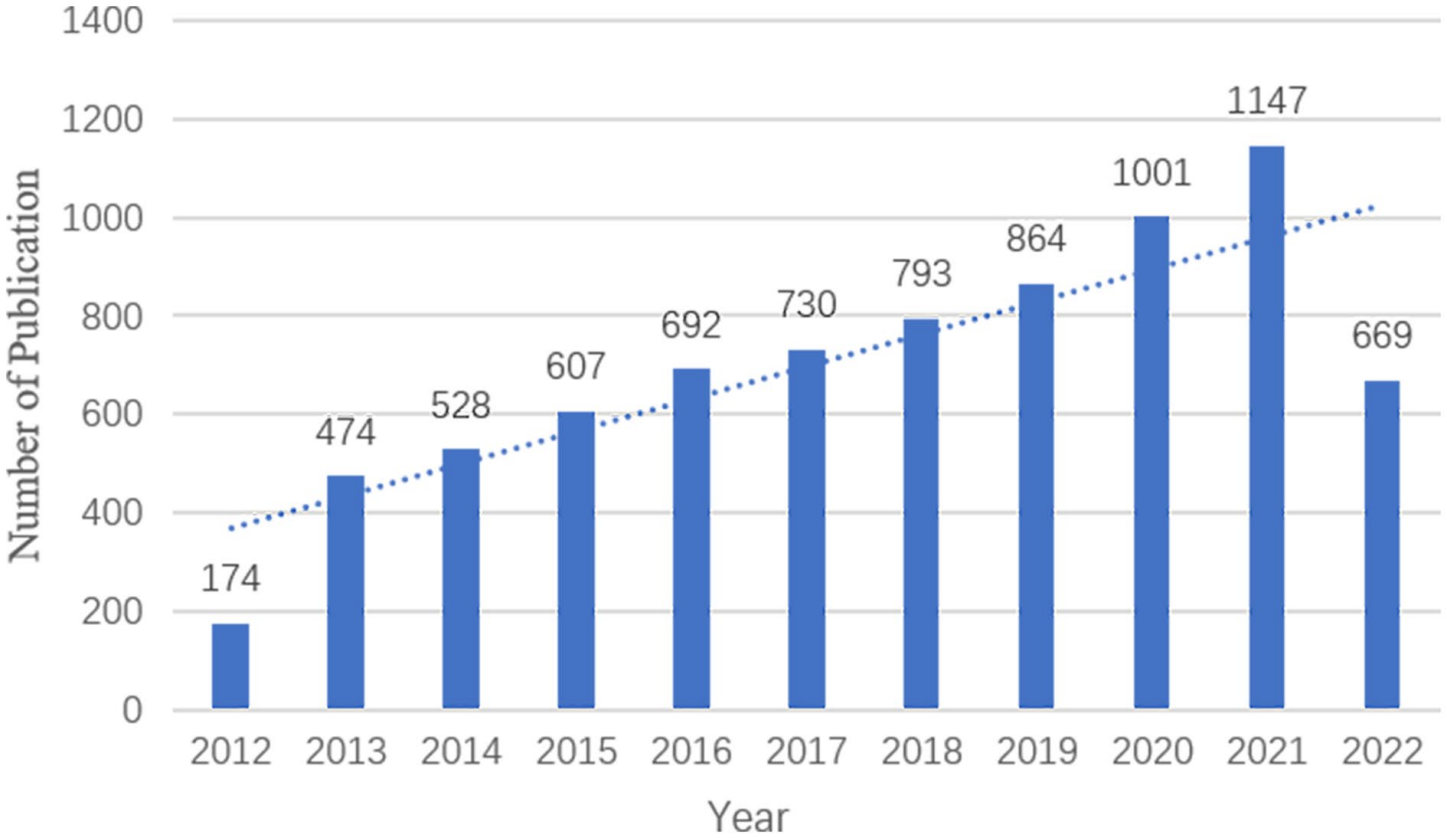
Distribution of annual publications.

Over the 10 years from 31 August 2012 to 31 August 2022, there were 7,679 publications relevant to imaging in sleep disorders, with a mean of 76.79 publications per year. There was an increasing trend in the number of publications per year from 2013 to 2021 (Figure 2).

### 3.2. Countries and institutions

**Figure 3:**
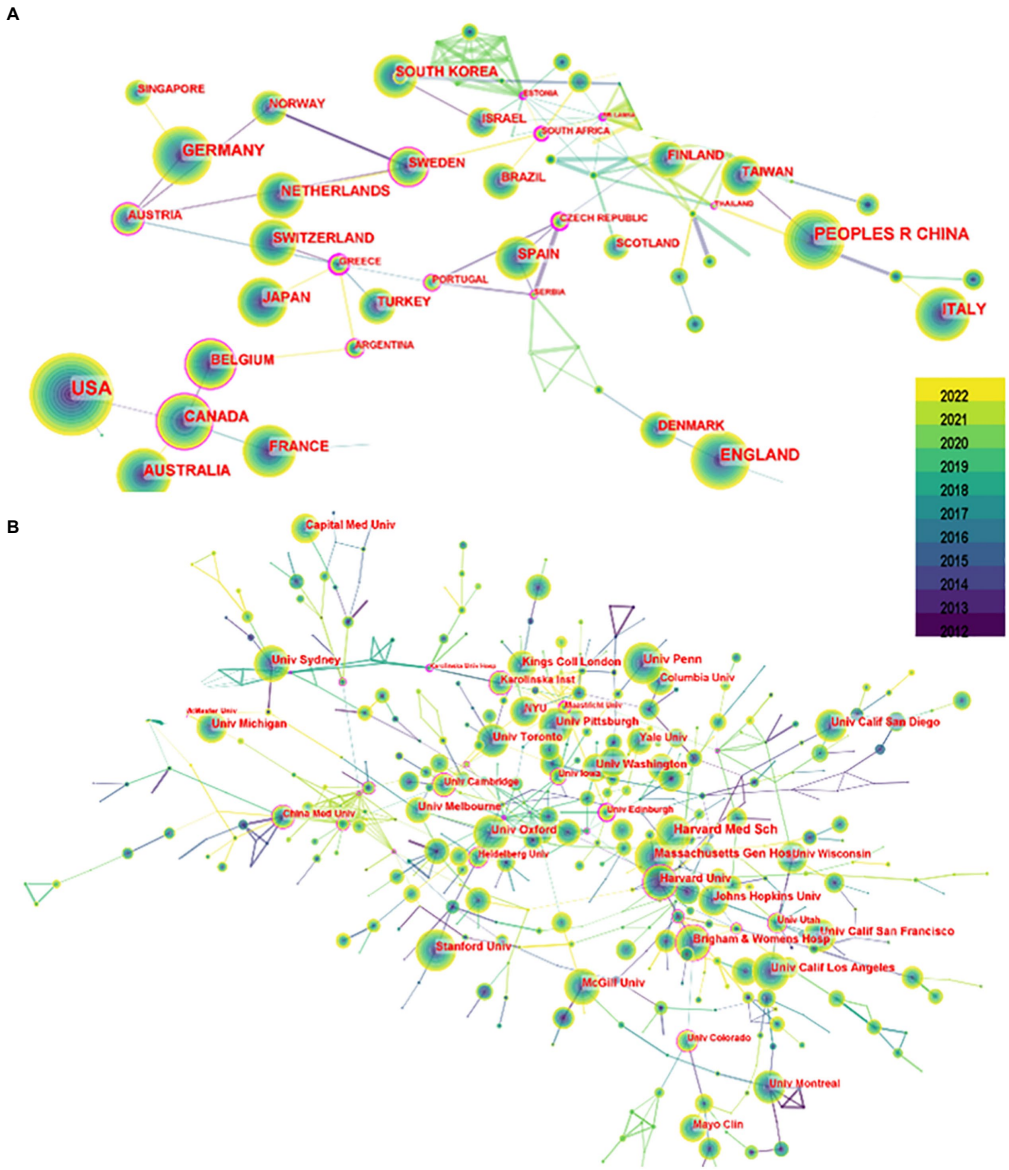
Mapping of issuing countries and institutions. **(A)** Shows the country/region from which publications issued, and **(B)** shows the issuing institution. Each circle in the figure represents a country, with the circle’s size indicating that country’s publication output. The lines connecting the circles represent international cooperation: the thicker the line, the closer the cooperation. The purple outer circle in the figure indicates centrality ≥0.10.

A total of 566 institutions from 135 countries published literature on imaging in sleep problems over the past decade. As measured by the number of publications, Table 1 provides a list of the top 10 countries/regions and institutions in the world. The United States (2,993/37.56%) and China (1,064/13.35%) had the most publications, accounting for more than half of the total. Among the top 10 institutions with the most publications, seven were in the United States (Table 1). Among the top 10 countries/regions and institutions in terms of volume, only Canada (0.12) and Harvard University (0.19) showed high centrality, as shown in Figure 3. There was less cooperation between countries, and regional cooperation between institutions appeared vital (Countries are represented by country co-authorship, and institutions are represented by institution co-authorship.).

**Table 1:** Top 10 countries/regions and institutions in terms of number of articles issued.

Ranking	Country/Region	Documents (%)	Centrality	Institution (country/region)	Documents (%)	Centrality
1	United States	2,993 (38.78%)	0.03	Harvard University (United States)	279 (3.63%)	0.19
2	China	1,064 (13.86%)	0.00	Birmingham Women’s Hospital (United Kingdom)	200 (2.60%)	0.16
3	United Kingdom	782 (10.18%)	0.03	University of Pennsylvania (United States)	183 (2.38%)	0.01
4	Germany	721 (9.39%)	0.03	University of California, Los Angeles (United States)	164 (2.14%)	0.00
5	Canada	557 (7.25%)	0.12	Stanford University (United States)	159 (2.07%)	0.01
6	Italy	545 (7.10%)	0.00	Harvard University (United States)	153 (1.99%)	0.19
7	Australia	537 (6.99%)	0.00	University of Sydney (Australia)	153 (1.99%)	0.05
8	France	475 (6.19%)	0.03	McGill University (Canada)	141 (1.84%)	0.04
9	Japan	438 (5.70%)	0.00	University of Pittsburgh (United States)	140 (1.82%)	0.01
10	Netherlands	350 (4.56%)	0.00	Johns Hopkins University (United States)	140 (1.82%)	0.01

### 3.3. Visual analysis of authors and co-cited authors

**Figure 4:**
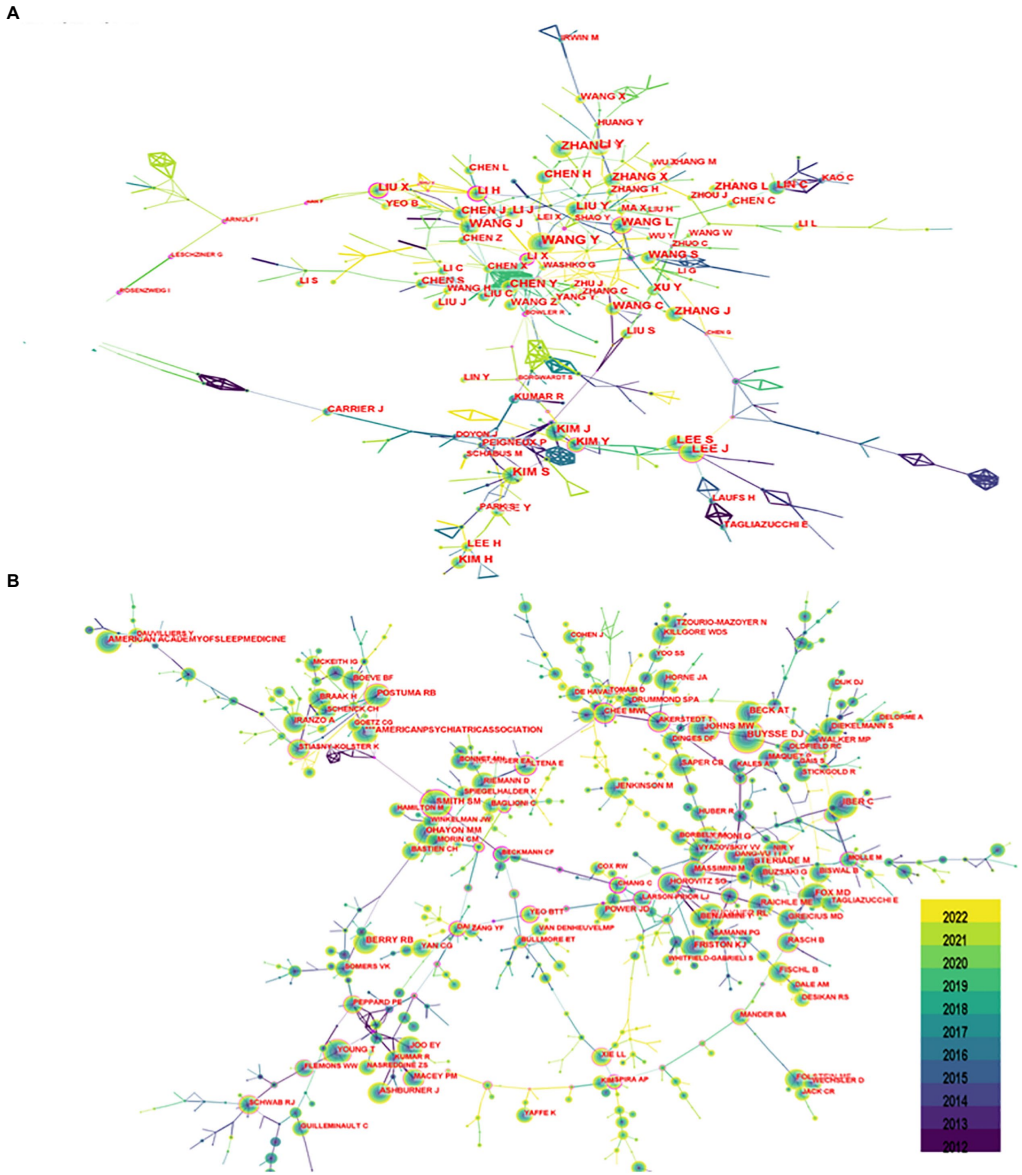
Author chart. **(A)** Shows the author graph, and **(B)** shows the co-cited author graph. The node size indicates the number of studies published or co-cited by the author, and a larger node suggests that the author has published more papers or co-cited more times. The closer the collaboration between two authors, the shorter the distance between the two nodes.

Seven hundred seventy-seven authors and 927 co-cited authors contributed to the study of imaging in sleep disorders. Among them, author Lee, J and co-citation authors Buysse, DJ (677); Smith, SM (403); Johns, MW (375); and Postuma, RB (289) had high centrality. Figure 4 shows communication and collaboration networks among the authors and co-cited authors in this study field, with collaborative exchanges increasing in recent years (Authors are represented by author co-authorship; Table 2)

**Table 2:** Top 10 authors and co-cited authors.

Ranking	Author	Documents	Centrality	Co-cited Authors	Citations	Centrality
1	Wang Y	125	0.02	Buysse DJ	677	0.14
2	Zhang Y	101	0.00	Iber C	405	0.00
3	Li Y	93	0.04	Smith SM	403	0.19
4	Lee J	90	0.12	Johns MW	375	0.14
5	Kim S	83	0.06	Berry RB	352	0.00
6	Wang J	82	0.03	Beck AT	297	0.00
7	Liu Y	78	0.08	Steriade M	293	0.08
8	Kim J	78	0.01	Fox MD	292	0.08
9	Lee S	76	0.00	Postuma RB	289	0.17
10	Wang L	71	0.06	Buckner RL	288	0.00

### 3.4. Journal analysis

There were 7,679 articles from 1,428 journals, among which *Sleep* was the journal with the largest number of publications (*n* = 265) and the most cited journal. Table 3 shows the top 10 journals and co-cited journals in the field of sleep-related imaging.

**Table 3:** Top 10 journals in terms of number of articles and citations.

Rank	Journal	Documents	JCR	Impact Factor	Cited Journal	Citations	JCR	Impact Factor
1	Sleep	265	Q1	6.316	Sleep	3,005	Q1	6.316
2	Scientific Reports	218	Q2	4.996	Plos One	2,995	Q2	3.752
3	Plos One	212	Q2	3.752	Neuroimage	2,634	Q1	7.400
4	Neuroimage	186	Q1	7.400	Proc Natl Acad Sci USA	2,504	Q1	12.779
5	Sleep Medicine	144	Q2	4.842	Journal of Neuroscience	2,471	Q1	6.709
6	Journal of Sleep Research	101	Q2	5.296	Neurology	2,057	Q1	11.800
7	Frontiers in Neurology	98	Q2	4.086	Brain	2,002	Q1	15.255
8	Frontiers in Neuroscience	97	Q2	5.152	Science	1,896	Q1	63.714
9	Journal of Neuroscience	89	Q1	6.709	Neuron	1,760	Q1	18.688
10	Cranio	86	Q4	1.670	Sleep Medicine	1,759	Q2	4.842

### 3.5. Keyword analysis

**Figure 5:**
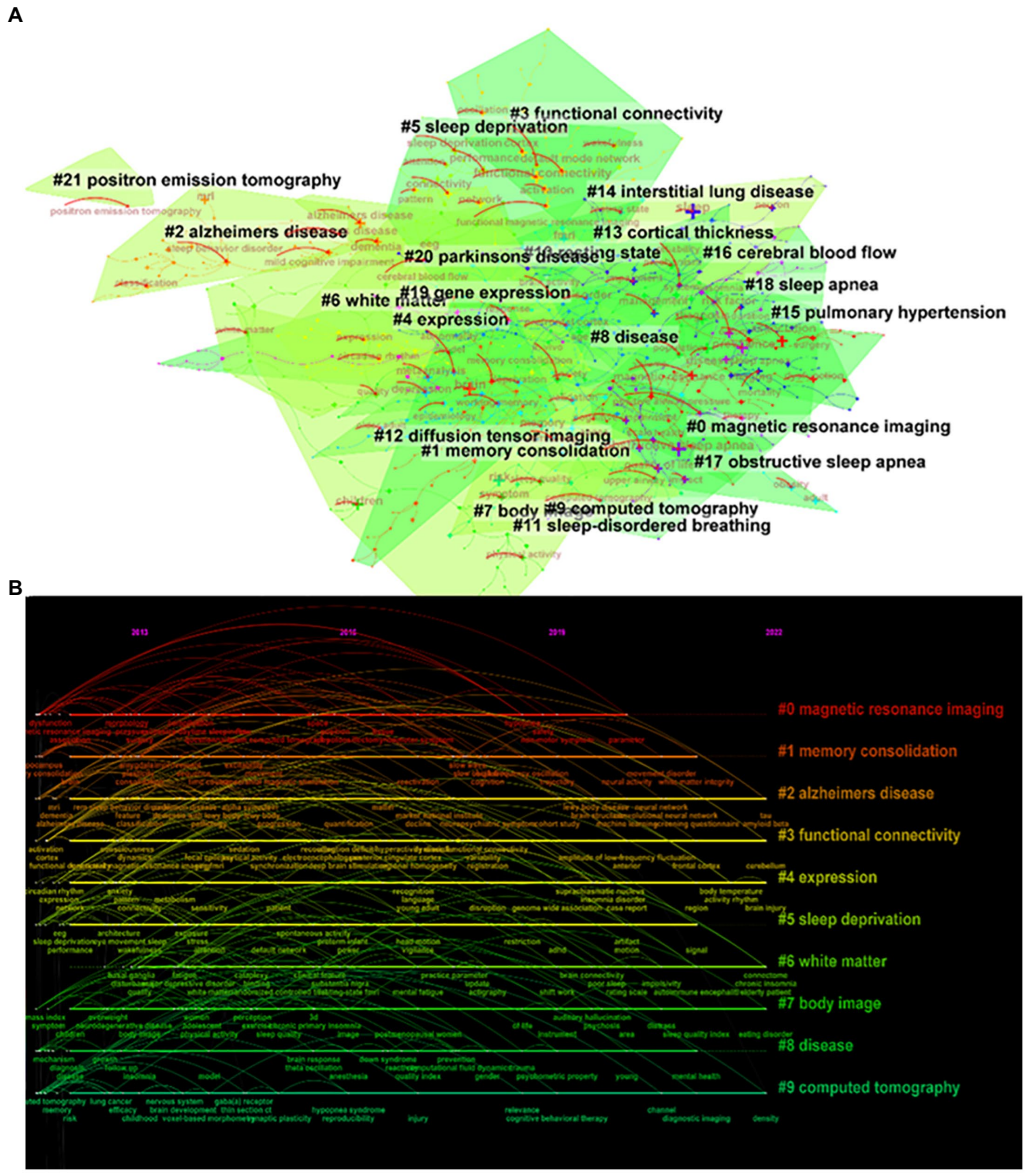
Keyword clustering analysis. **(A)** Keyword clustering mapping, **(B)** Keyword clustering timeline mapping.

**Figure 6:**
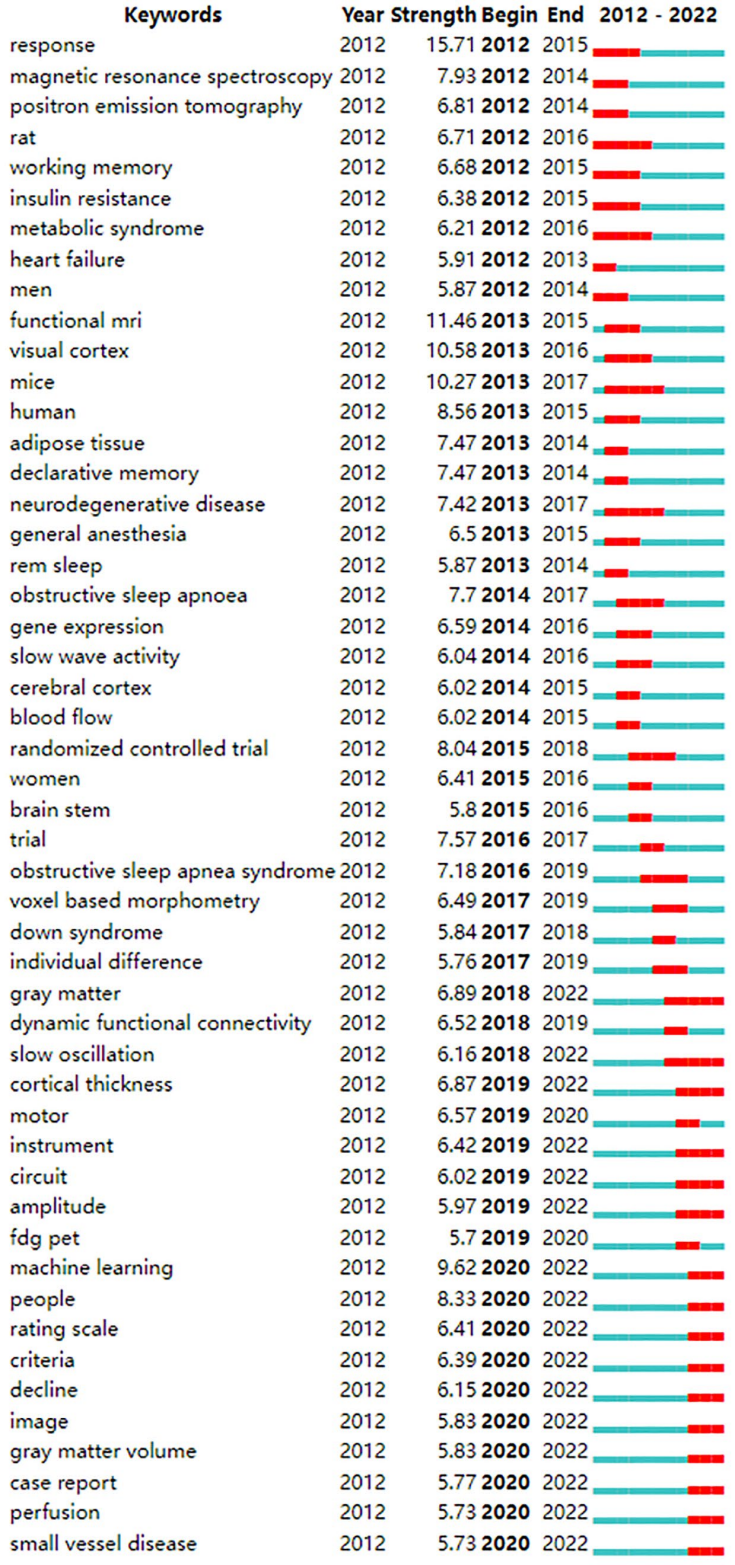
Keyword strongest citation bursts map.

From the keyword co-occurrence map obtained by CiteSpace, we extracted a total of 662 keywords. The top 20 keywords with the highest frequency are shown in Table 4, indicating the hotspots of imaging applications in the field of sleep. The diseases most frequently studied were obstructive sleep apnea, Parkinson’s disease, and Alzheimer’s disease. Clustering of keywords revealed a cluster Q value of 0.8342 (Q > 0.3) and S value of 0.9352 (S > 0.5), showing that the clustering structure was significant and reasonable. Based on keyword clustering analysis, 22 clusters were formed (Figure 5A), from which we extracted the top 10 clusters in size for timeline analysis (Figure 5B). Imaging studies of sleep predominately use magnetic resonance imaging. The keyword has been popular since 2012, including functional imaging, white matter visualization, and body imaging. Memory consolidation is the function of greatest concern to researchers, and the condition of greatest concern is Alzheimer’s disease. While interest in sleep deprivation has waned in recent years, research on emotion has received increasing attention. Among the keywords in the top 50 strongest citation burst (Figure 6), those representing the frontiers of research were machine learning, people, rating scales, criteria, decline, images, gray matter volume, case reports, perfusion, and small vessel disease, with machine learning having the highest emergent intensity.

**Table 4:** Top 20 keywords in terms of frequency of appearance.

Rank	Keywords	Frequency	Rank	Keywords	Frequency
1	Sleep	764	11	Disease	327
2	Obstructive sleep apnea	639	12	Alzheimer’s disease	325
3	Brain	522	13	Diagnosis	300
4	Functional connectivity	486	14	Performance	299
5	Children	420	15	Cortex	259
6	Risk	395	16	Network	257
7	Prevalence	384	17	Magnetic resonance imaging	256
8	Association	366	18	Sleep deprivation	253
9	Parkinson disease	343	19	Dementia	253
10	Disorder	340	20	Memory	248

### 3.6. Co-cited reference analysis

**Figure 7:**
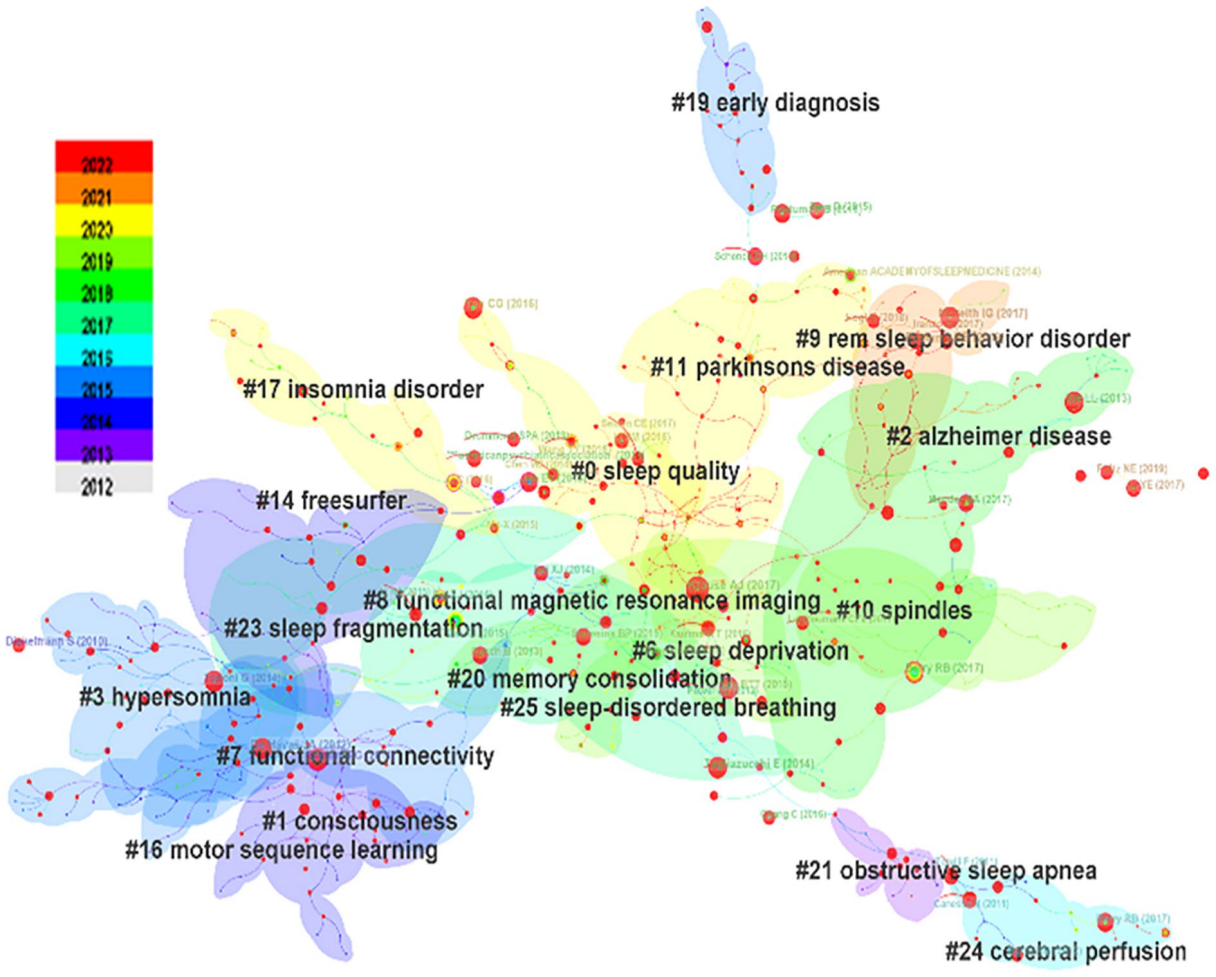
Co-citation clustering map of the literature.

The clustering structure was significant and the clustering content was convincing. Seven thousand six hundred seventy-nine citing articles were analyzed to identify homogeneous clusters of highly cited literature on sleep-related imaging studies. Table 4 shows the top 10 most co-cited references out of 225,108 co-cited references. “*The sleep-deprived human brain*” (Krause et al., 2017) published by Adam J. Krause in the journal Nature Reviews Neuroscience in 2017, was the most frequently cited publication, with 80 citations. The co-cited literature was subjected to cluster analysis, which generated a cluster Q value (module value) of 0.9051 (Q > 0.3) and an S value (mean profile value) of 0.9597 (S > 0.7), signifying a significant cluster structure and convincing cluster content (Figure 7; Table 5).

**Table 5:** Top 10 literature in terms of total citations.

Rank	Cited reference	Journal	Author	citations	Centrality	Year
1	The sleep-deprived human brain	Nat Rev. Neurosci	Adam J. Krause	80	0.05	2017
2	Decoding wakefulness levels from typical fMRI resting-state data reveal reliable drifts between wakefulness and sleep	Neuron	Enzo Tagliazucchi	69	0	2014
3	Diagnosis and management of dementia with Lewy bodies: Fourth consensus report of the DLB Consortium	Neurology	Ian G. McKeith	65	0.01	2017
4	Risk and predictors of dementia and Parkinsonism in idiopathic REM sleep behavior disorder: a multicenter study	Brain	Ronald B. Postuma	63	0.03	2019
5	Sleep deprivation reduces default mode network connectivity and anti-correlation during rest and task performance	Neuroimage	Jack A. De Havas	60	0.09	2012
6	DPABI: Data Processing and Analysis for (Resting-State) Brain Imaging	Neuroinformatics	Chao-Gan Yan	59	0	2016
7	Sleep drives metabolite clearance from the adult brain	Science	Lulu Xie	57	0.02	2013
8	AASM Scoring Manual Updates for 2017 (Version 2.4)	J Clin Sleep Med	Richard B. Berry	56	0.01	2017
9	The memory function of sleep	Nat Rev. Neurosci	Susanne Diekelmann	52	0.04	2010
10	MDS clinical diagnostic criteria for Parkinson’s disease	Movement Disorder	Ronald B. Postuma	50	0	2015

## 4. Discussion

In this study, WOSCC was searched for publications related to imaging in sleep disorders in the past 10 years. The search results were analyzed using CiteSpace software. The number of articles published in the past 10 years has increased on a yearly basis, and imaging has received increasing attention in sleep research. The top international publications have been from developed nations in Europe and the United States, particularly the latter. European and American research institutions, headed by Harvard University, have conducted more in-depth research and made more significant contributions to this field than other locations or institutions. Developing countries have not paid sufficient attention to this field, lack influential institutions, and there is room for their development. Cooperation among institutions shows regionalization, with relatively less international multi-center cooperation. International cooperation could be strengthened and more meaningful research could be carried out in the future. China had the second largest number of published articles, and Chinese persons authored the most articles in this field, which may be because China has a large population. The author analysis of CiteSpace is not based on the first author but on all participating authors. However, there is a considerable problem for Chinese researchers: they have published many articles, but have received few citations. That is, their research results relatively lack international recognition. For Chinese scholars, it is crucial to conduct more valuable, high-quality research. Increasing international influence is essential. In the analysis of co-citations of journals, *Sleep* had the largest number of articles and co-citations in this field and is the most influential journal, which is of guiding significance for researchers when reading the literature and publishing results. As can be seen from the keyword time chart, with the development of imaging technology and computer technology, the research on sleep is becoming more and more elaborate, and the computer post-processing technology brings great changes to the research, so we can make a quantitative analysis of imaging indicators. And the research extends from organic pathological changes to psychological cognition, from macro to micro.

According to keyword analysis, research has focused on the effects of degenerative diseases on sleep, such as Parkinson’s disease (Postuma et al., 2015; Krause et al., 2017; McKeith et al., 2017; Postuma et al., 2019), Alzheimer’s disease (Mizrahi-Kliger et al., 2022; Sun et al., 2022), and small vessel disease (Semyachkina-Glushkovskaya et al., 2020; Li et al., 2022). We speculate that this may be related to the aging of the population and the fact that the elderly are more likely to develop sleep disorders. It is reported that insomnia is a common sleep disorder in the elderly, of which up to 50% report insomnia symptoms, and according to the diagnostic guidelines used, up to 20% of patients meet the criteria for insomnia (Sexton et al., 2020). The incidence and prevalence of sleep disorders continues to in older persons, especially those with neurodegenerative diseases. A recent study clarified the mechanisms of neurodegeneration exacerbated by poor sleep quality and circadian rhythm disorders (Standlee and Malkani, 2022). Small vessel disease exists in both Parkinson’s and Alzheimer’s diseases (Paolini Paoletti et al., 2021); this refers to the dominant injury of arterioles and capillaries, resulting in reduced or interrupted perfusion of the affected organs, primarily affecting organs that receive most of the cardiac output, such as the brain, kidney, and retina (Hakim, 2019). Sleep disorders can adversely affect the regulation of gene expression or protein production, resulting in pathological protein changes. The accumulation of toxic proteins in Parkinson’s and Alzheimer’s diseases, such as alpha-synuclein, TDP-43, A, β, and tau, has been shown to disrupt the sleep-awakening cycle (Dong et al., 2019). Sleep disorders promote the development of neurodegenerative symptoms (Lucey et al., 2018), increasing the risk of Parkinson’s and Alzheimer’s disease and aggravating the symptoms of these diseases. Such symptoms include muscle stiffness, tremor, motor dysfunction, and cognitive impairment, among which researchers are most concerned with the patient’s cognitive ability, especially memory ability (Figure 6). This may be due to the effect of sleep on cognitive ability has been proved by several studies (Dzierzewski et al., 2018; Ma et al., 2020). For the elderly, the decline of memory ability leads to a serious decline in the quality of life, which is the most easily detected symptom in life.

Symptom assessment is very important for diagnosing and treating neurological diseases, not only in clinical practice but also in basic research. It is usually necessary to observe the severity of neurological diseases and use appropriate methods to evaluate the effectiveness of specific treatments (Asakawa et al., 2019). There is a growing demand for cognitive assessment tools in the field of sleep imaging; accordingly, assessment scales and criteria have become popular keywords in the previous 2 years. Magnetic resonance functional brain imaging (De Havas et al., 2012; Tagliazucchi and Laufs, 2014; Yan et al., 2016) and the development of image processing software provide objective and quantitative evaluation tools for application in sleep research. The development of MRI techniques and data processing technologies that have emerged in the previous decade have permitted accurate assessment of brain structure, function, and metabolism for sleep research, and imaging has provided new tools for the development of this field. As the keyword with the strongest citation bursts after 2020, machine learning is both a subfield of artificial intelligence and a method for artificial intelligence research and development. It uses a combination of mathematical, statistical, probabilistic, and information-theoretic methods to learn and tune performance on specific tasks, using large, and real-world data sets. Artificial intelligence is rapidly advancing in the field of imaging, with a wide range of applications in the screening, staging, diagnosis, and treatment of sleep disorders (Watson and Fernandez, 2021).

The most recent four clusters cited in the literature encompass Parkinson’s disease, insomnia, sleep quality, and REM sleep behavior disorder (Figure 5), namely these topics were cited the most. In the field of sleep research, Parkinson’s disease is the condition of greatest concern among diseases that cause sleep disorders. Conditions of most concern are insomnia and REM sleep behavior disorder, a type of abnormal sleep considered to be the precursor to α-synaptophysin disease, such as Parkinson’s disease; such disordered sleep affects more than 50% of patients with Parkinson’s disease (Valli et al., 2022). In addition, insomnia is one of the most common sleep disorders in Parkinson’s disease and is significantly associated with poor quality of life (Diaconu and Falup-Pecurariu, 2022).

Recent studies have revealed that the neural mechanisms responsible for these sleep disorders may be related to abnormalities and interhemispheric interactions in brain regions associated with excessive arousal and sensorimotor and cognition. Imaging in such cases is characterized by topological organization disorder of functionally connected brain groups, which may lead to decreased cognitive, emotional, and memory function (Fasiello et al., 2022). Further, sleep disorders are associated with various changes in magnetic resonance functional brain imaging, such as gray matter volume (Paulekiene et al., 2022), cortical thickness (Babu Henry Samuel et al., 2022), and white matter function (Bai et al., 2022; Yang et al., 2022). These indexes reflect changes in brain structure, function, and metabolism, and provide a qualitative and quantitative basis for the study of sleep disorders, and guidance for disease prediction and future treatment target selection, thus may provide suggestions as to how to relieve patients’ pain and provide early diagnosis and treatment of related diseases.

The primary deficiency of this study is that the scope of literature was limited to the WOS database, with the exclusion of PubMed, Embase, and other databases. As the relationship between co-citations is analyzed, only the citation database WoSCC can be selected, which may limit the inclusion of literature. This may have permitted bias in the results, but the WOS database, as the most cited database, cover most of the high-quality sleep imaging literature. And CiteSpace software evaluates countries, institutions, and authors based on all co authored countries, co-authored institutions, and co-authors, hence it is unable to differentiate between the first author and other authors.

Overall, the analysis using CiteSpace software showed that the focus of research attention is the impact of degenerative diseases on sleep. With population aging, sleep disorders and diminished sleep quality caused by degenerative diseases are receiving increasing attention. Sleep efficiency, slow wave sleep volume, REM sleep, and REM sleep latency all decrease with increasing age (Etholén et al., 2022). With the rapid development of magnetic resonance software and hardware technology, sleep imaging will likely continue to provide strong support for research into sleep disorders caused by aging. The current research is becoming increasingly detailed. This study shows a trend in imaging of sleep in degenerative disease research from symptoms to changes in brain structure and function in diseases that cause sleep disorders and changes in sleep quality, with the primary goal of predicting the occurrence of sleep disorders in degenerative disease patients.

## Data availability statement

The original contributions presented in the study are included in the article/supplementary material, further inquiries can be directed to the corresponding author.

## Author contributions

ML and ZJ mainly focused on data collection, data analysis, and writing. CL and RW contributed to manuscript revision. JW and CL contributed to manuscript reviewing and suggestion and contributed to the conception, supervision, and reviewing. All authors contributed to the article and approved the submitted version.

## Funding

This work was supported by National Natural Science Foundation of China (grant number 82071910 and 81601478).

## Conflict of interest

The authors declare that the research was conducted in the absence of any commercial or financial relationships that could be construed as a potential conflict of interest.

## Publisher’s note

All claims expressed in this article are solely those of the authors and do not necessarily represent those of their affiliated organizations, or those of the publisher, the editors and the reviewers. Any product that may be evaluated in this article, or claim that may be made by its manufacturer, is not guaranteed or endorsed by the publisher.

## Footnotes

^1^ https://sourceforge.net/projects/citespace/

^2^ https://www.microsoft.com/en-us/microsoft-365/previous-versions/microsoft-office-2019

## References

[ref1] Abbas A. M. (2012). Bounds and inequalities relating h-index, g-index, e-index and generalized impact factor: an improvement over existing models. PLoS One 7:e33699. doi: 10.1371/journal.pone.0033699, PMID: 22496760 PMC3319552

[ref2] Asakawa T. Sugiyama K. Nozaki T. Sameshima T. Kobayashi S. Wang L.. (2019). Can the latest computerized technologies revolutionize conventional assessment tools and therapies for a neurological disease? The example of Parkinson's disease. Neurol. Med. Chir. 59, 69–78. doi: 10.2176/nmc.ra.2018-0045, PMID: 30760657 PMC6434424

[ref3] Assenov Y. Ramírez F. Schelhorn S. E. Lengauer T. Albrecht M. (2008). Computing topological parameters of biological networks. Bioinformatics 24, 282–284. doi: 10.1093/bioinformatics/btm554 18006545

[ref4] Babu Henry Samuel I. Pollin K. U. Breneman C. B. (2022). Lower cortical volume is associated with poor sleep quality after traumatic brain injury. Brain Imag. Behav. 16, 1362–1371. doi: 10.1007/s11682-021-00615-4 35018551

[ref5] Bai Y. Zhang L. Liu C. Cui X. Li D. Yin H. (2022). Association of white matter volume with sleep quality: a voxel-based morphometry study. Brain Imag. Behav. 16, 1163–1175. doi: 10.1007/s11682-021-00569-7, PMID: 34846693

[ref6] Bin Heyat M. B. Akhtar F. Ansari M. A. Khan A. Alkahtani F. Khan H.. (2021). Progress in detection of insomnia sleep disorder: a comprehensive review. Curr. Drug Targets 22, 672–684. doi: 10.2174/1389450121666201027125828, PMID: 33109045

[ref7] Bourgouin P. A. Rahayel S. Gaubert M. Arnaldi D. Hu M. Heidbreder A.. (2019). Neuroimaging of rapid eye movement sleep behavior disorder. Int. Rev. Neurobiol. 144, 185–210. doi: 10.1016/bs.irn.2018.10.006 30638454

[ref8] Chen C. Dubin R. Kim M. C. (2014). Emerging trends and new developments in regenerative medicine: a scientometric update (2000-2014). Expert. Opin. Biol. Ther. 14, 1295–1317. doi: 10.1517/14712598.2014.920813, PMID: 25077605

[ref9] Chen C. Song M. (2019). Visualizing a field of research: a methodology of systematic scientometric reviews. PLoS One 14:e0223994. doi: 10.1371/journal.pone.0223994, PMID: 31671124 PMC6822756

[ref10] De Havas J. A. Parimal S. Soon C. S. Chee M. W. (2012). Sleep deprivation reduces default mode network connectivity and anti-correlation during rest and task performance. NeuroImage 59, 1745–1751. doi: 10.1016/j.neuroimage.2011.08.026, PMID: 21872664

[ref11] Diaconu Ş. Falup-Pecurariu C. (2022). Personalized assessment of insomnia and sleep quality in patients with Parkinson's disease. J. Personal. Med. 12:322. doi: 10.3390/jpm12020322, PMID: 35207811 PMC8875986

[ref12] Ding H. Wu C. Liao N. Zhan Q. Sun W. Huang Y.. (2021). Radiomics in oncology: a 10-year bibliometric analysis. Front. Oncol. 11:689802. doi: 10.3389/fonc.2021.689802, PMID: 34616671 PMC8488302

[ref13] Dong H. Wang J. Yang Y. F. Shen Y. Qu W. M. Huang Z. L. (2019). Dorsal striatum dopamine levels fluctuate across the sleep-wake cycle and respond to salient stimuli in mice. Front. Neurosci. 13:242. doi: 10.3389/fnins.2019.00242, PMID: 30949023 PMC6436203

[ref14] Dzierzewski J. M. Dautovich N. Ravyts S. (2018). Sleep and cognition in older adults. Sleep Med. Clin. 13, 93–106. doi: 10.1016/j.jsmc.2017.09.009, PMID: 29412987 PMC5841581

[ref15] Etholén A. Pietiläinen O. Kouvonen A. Hänninen M. Rahkonen O. Lallukka T. (2022). Trajectories of insomnia symptoms among aging employees and their associations with memory, learning ability, and concentration after retirement - a prospective cohort study (2000-2017). J. Aging Health 34, 916–928. doi: 10.1177/08982643221078740, PMID: 35482013 PMC9483690

[ref16] Fasiello E. Gorgoni M. Scarpelli S. Alfonsi V. Ferini Strambi L. De Gennaro L. (2022). Functional connectivity changes in insomnia disorder: a systematic review. Sleep Med. Rev. 61:101569. doi: 10.1016/j.smrv.2021.101569, PMID: 34902821

[ref17] Ferini-Strambi L. Fasiello E. Sforza M. Salsone M. Galbiati A. (2019). Neuropsychological, electrophysiological, and neuroimaging biomarkers for REM behavior disorder. Expert. Rev. Neurother. 19, 1069–1087. doi: 10.1080/14737175.2019.1640603, PMID: 31277555

[ref18] Glanzel W. (2015). Bibliometrics-aided retrieval: where information retrieval meets scientometrics. Scientometrics 102, 2215–2222. doi: 10.1007/s11192-014-1480-7

[ref19] Hakim A. M. (2019). Small vessel disease. Front. Neurol. 10:1020. doi: 10.3389/fneur.2019.01020, PMID: 31616367 PMC6768982

[ref20] Hou J. H. Yang X. C. Chen C. M. (2018). Emerging trends and new developments in information science: a document co-citation analysis (2009-2016). Scientometrics 115, 869–892. doi: 10.1007/s11192-018-2695-9

[ref21] Krause A. J. Simon E. B. Mander B. A. Greer S. M. Saletin J. M. Goldstein-Piekarski A. N.. (2017). The sleep-deprived human brain. Nat. Rev. Neurosci. 18, 404–418. doi: 10.1038/nrn.2017.55, PMID: 28515433 PMC6143346

[ref22] Li X. Qin R. R. Chen J. Jiang H. F. Tang P. Wang Y. J.. (2022). Neuropsychiatric symptoms and altered sleep quality in cerebral small vessel disease. Front. Psychol. 13:882922. doi: 10.3389/fpsyt.2022.882922, PMID: 36051552 PMC9424898

[ref23] Lucey B. P. Hicks T. J. McLeland J. S. Toedebusch C. D. Boyd J. Elbert D. L.. (2018). Effect of sleep on overnight cerebrospinal fluid amyloid β kinetics. Ann. Neurol. 83, 197–204. doi: 10.1002/ana.25117, PMID: 29220873 PMC5876097

[ref24] Ma Y. Liang L. Zheng F. Shi L. Zhong B. Xie W. (2020). Association between sleep duration and cognitive decline. JAMA Netw. Open 3:e2013573. doi: 10.1001/jamanetworkopen.2020.13573, PMID: 32955572 PMC7506513

[ref25] Malkani R. G. Zee P. C. (2022). Brain stimulation for improving sleep and memory. Sleep Med. Clin. 17, 505–521. doi: 10.1016/j.jsmc.2022.06.013, PMID: 36150810

[ref26] McKeith I. G. Boeve B. F. Dickson D. W. Halliday G. Taylor J. P. Weintraub D.. (2017). Diagnosis and management of dementia with Lewy bodies: fourth consensus report of the DLB consortium. Neurology 89, 88–100. doi: 10.1212/WNL.0000000000004058, PMID: 28592453 PMC5496518

[ref27] Mizrahi-Kliger A. D. Feldmann L. K. Kühn A. A. Bergman H. (2022). Etiologies of insomnia in Parkinson's disease - lessons from human studies and animal models. Exp. Neurol. 350:113976. doi: 10.1016/j.expneurol.2022.113976, PMID: 35026228

[ref28] Paolini Paoletti F. Simoni S. Parnetti L. Gaetani L. (2021). The contribution of small vessel disease to neurodegeneration: focus on Alzheimer's disease, Parkinson's disease and multiple sclerosis. Int. J. Mol. Sci. 22:4958. doi: 10.3390/ijms22094958, PMID: 34066951 PMC8125719

[ref29] Paulekiene G. Pajarskiene M. Pajediene E. Radziunas A. (2022). Sleep dysfunction and Grey matter volume. Curr. Neurol. Neurosci. Rep. 22, 275–283. doi: 10.1007/s11910-022-01190-x, PMID: 35364772

[ref30] Postuma R. B. Berg D. Stern M. Poewe W. Olanow C. W. Oertel W.. (2015). MDS clinical diagnostic criteria for Parkinson's disease. Mov. Disord. 30, 1591–1601. doi: 10.1002/mds.26424 26474316

[ref31] Postuma R. B. Iranzo A. Hu M. Högl B. Boeve B. F. Manni R.. (2019). Risk and predictors of dementia and parkinsonism in idiopathic REM sleep behaviour disorder: a multicentre study. Brain 142, 744–759. doi: 10.1093/brain/awz030, PMID: 30789229 PMC6391615

[ref32] Rundo J. V. Downey R. 3rd (2019). Polysomnography. Handb. Clin. Neurol. 160, 381–392. doi: 10.1016/B978-0-444-64032-1.00025-4 31277862

[ref33] Sabe M. Chen C. Sentissi O. Deenik J. Vancampfort D. Firth J.. (2022). Thirty years of research on physical activity, mental health, and wellbeing: a scientometric analysis of hotspots and trends. Front. Public Health 10:943435. doi: 10.3389/fpubh.2022.943435, PMID: 36016904 PMC9396383

[ref34] Schulz H. (2022). The history of sleep research and sleep medicine in Europe. J. Sleep Res. 31:e13602. doi: 10.1111/jsr.13602 35522132

[ref35] Semyachkina-Glushkovskaya O. Postnov D. Penzel T. Kurths J. (2020). Sleep as a novel biomarker and a promising therapeutic target for cerebral small vessel disease: a review focusing on Alzheimer's disease and the blood-brain barrier. Int. J. Mol. Sci. 21:6293. doi: 10.3390/ijms21176293, PMID: 32878058 PMC7504101

[ref36] Sexton C. E. Sykara K. Karageorgiou E. Zitser J. Rosa T. Yaffe K.. (2020). Connections between insomnia and cognitive aging. Neurosci. Bull. 36, 77–84. doi: 10.1007/s12264-019-00401-9, PMID: 31222500 PMC6940406

[ref37] Spaggiari G. Romeo M. Casarini L. Granata A. R. M. Simoni M. Santi D. (2022). Human fertility and sleep disturbances: a narrative review. Sleep Med. 98, 13–25. doi: 10.1016/j.sleep.2022.06.009, PMID: 35772248

[ref38] Standlee J. Malkani R. (2022). Sleep dysfunction in movement disorders: a window to the disease biology. Curr. Neurol. Neurosci. Rep. 22, 565–576. doi: 10.1007/s11910-022-01220-8, PMID: 35867306

[ref39] Sun Y. Y. Wang Z. Zhou H. Y. Huang H. C. (2022). Sleep-wake disorders in Alzheimer's disease: a review. ACS Chem. Neurosci. 13, 1467–1478. doi: 10.1021/acschemneuro.2c00097 35507669

[ref40] Tagliazucchi E. Laufs H. (2014). Decoding wakefulness levels from typical fMRI resting-state data reveals reliable drifts between wakefulness and sleep. Neuron 82, 695–708. doi: 10.1016/j.neuron.2014.03.020, PMID: 24811386

[ref41] Tibon R. Tsvetanov K. A. (2021). The neural shift of sleep quality and cognitive aging: a resting-state MEG study of transient neural dynamics. Front. Aging Neurosci. 13:746236. doi: 10.3389/fnagi.2021.746236 35173599 PMC8842663

[ref42] Valli M. Uribe C. Mihaescu A. Strafella A. P. (2022). Neuroimaging of rapid eye movement sleep behavior disorder and its relation to Parkinson's disease. J. Neurosci. Res. 100, 1815–1833. doi: 10.1002/jnr.25099, PMID: 35790021

[ref43] Watson N. F. Fernandez C. R. (2021). Artificial intelligence and sleep: advancing sleep medicine. Sleep Med. Rev. 59:101512. doi: 10.1016/j.smrv.2021.101512 34166990

[ref44] Xu S. Faust O. Seoni S. Chakraborty S. Barua P. D. Loh H. W.. (2022). A review of automated sleep disorder detection. Comput. Biol. Med. 150:106100. doi: 10.1016/j.compbiomed.2022.106100, PMID: 36182761

[ref45] Yan C. G. Wang X. D. Zuo X. N. Zang Y. F. (2016). DPABI: Data Processing & Analysis for (resting-state) brain imaging. Neuroinformatics 14, 339–351. doi: 10.1007/s12021-016-9299-4, PMID: 27075850

[ref46] Yang Y. Wang S. Liu J. Zou G. Jiang J. Jiang B.. (2022). Changes in white matter functional networks during wakefulness and sleep. Hum. Brain Mapp. 43, 4383–4396. doi: 10.1002/hbm.25961, PMID: 35615855 PMC9435017

